# Supplementary material for: Unveiling chromatin dynamics with virtual epigenome
Source: Nat Commun. 2025 Apr 12;16:3491. doi: 10.1038/s41467-025-58481-3 (PMC11993739; doi:10.1038/s41467-025-58481-3)
Supplement: Supplementary file 1 — Supplementary Information [file 41467_2025_58481_MOESM1_ESM.pdf]

Supplementary Information for:

Unveiling Chromatin Dynamics with Virtual Epigenome

Ming-Yu Lin[1], Yu-Cheng Lo[1], and Jui–Hung Hung[1,2*]

[1] Department of Computer Science, National Yang Ming Chiao Tung University, HsinChu, Taiwan, ROC
[2] Program in Biomedical Artificial Intelligence, National Tsing Hua University, HsinChu, Taiwan, ROC

[*] To whom correspondence should be addressed. Email: jhh@cs.nycu.edu.tw or juihunghung@gmail.com

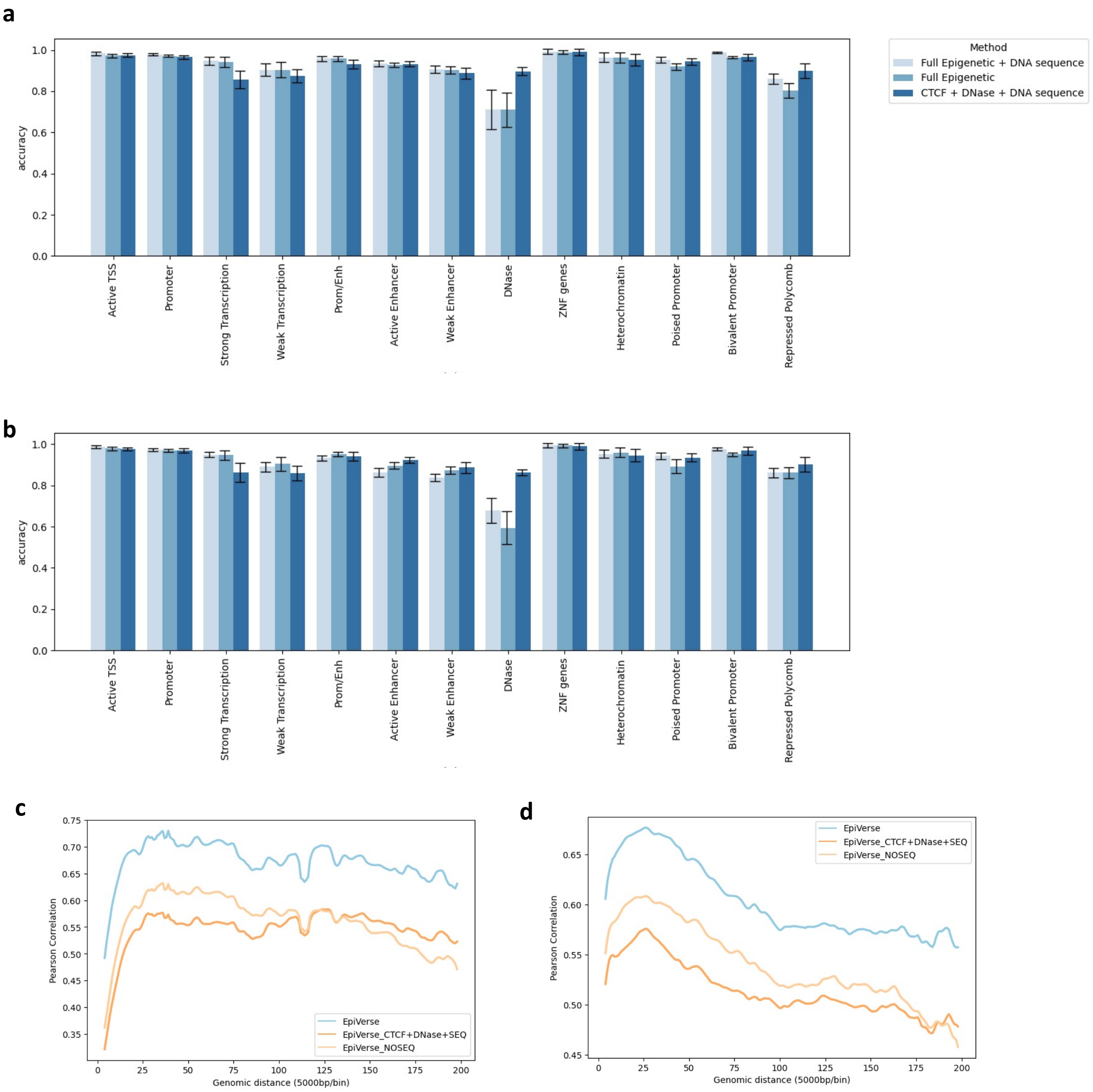

Supplementary Figure 1 EpiVerse Model Ablation Analysis.

a. ChromHMM Prediction Accuracy in GM12878: Depicts the accuracy of chromatin state predictions in GM12878 cells using the IMR90-trained EpiVerse model. Error bars indicate the standard deviations of accuracy (n=10,159). **b. ChromHMM Prediction Accuracy in K562:** Showcases the performance of the IMR90-trained EpiVerse model in accurately predicting chromatin states in K562 cells (n=10,159). Error bars indicate the standard deviations of accuracy. **c. Distance-Stratified Correlation in GM12878:** Presents the distance-stratified correlation scores for GM12878 predictions made by the IMR90-trained EpiVerse model. **d. Distance-Stratified Correlation in K562:** Illustrates the distance-stratified correlation scores for K562 predictions made by the IMR90-trained EpiVerse model, demonstrating the model's efficacy in capturing

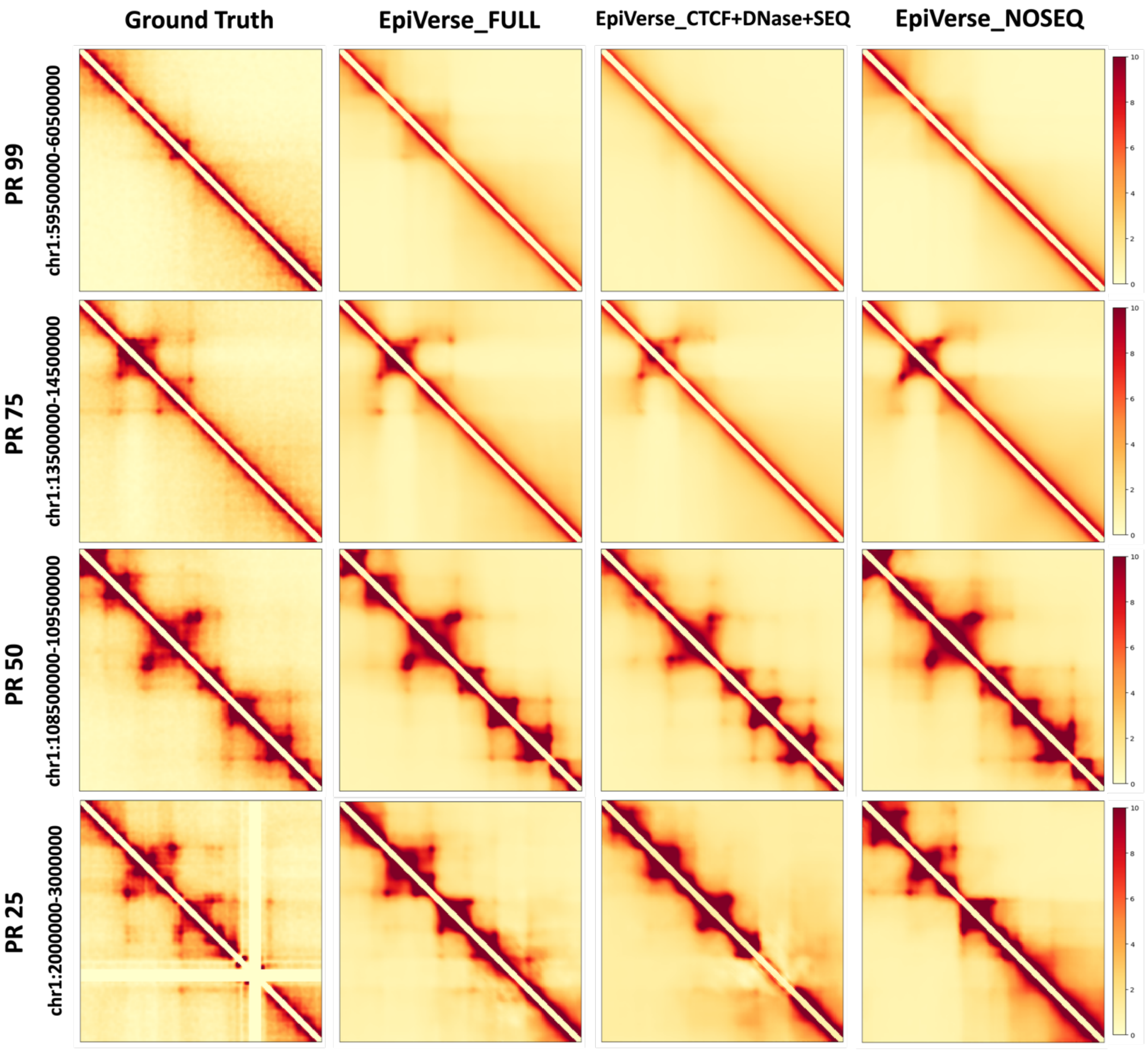

Supplementary Figure 2 Comparative Hi-C visualization from EpiVerse model ablations.

Columns from left to right display the contact maps generated from different model configurations. Rows categorize the Hi-C maps by the percentile ranks of each method's predictions, offering a comparative view of model performance across varying prediction ranks. The full setting consistently demonstrates the highest fidelity in chromatin structure predictions across all percentile ranks.

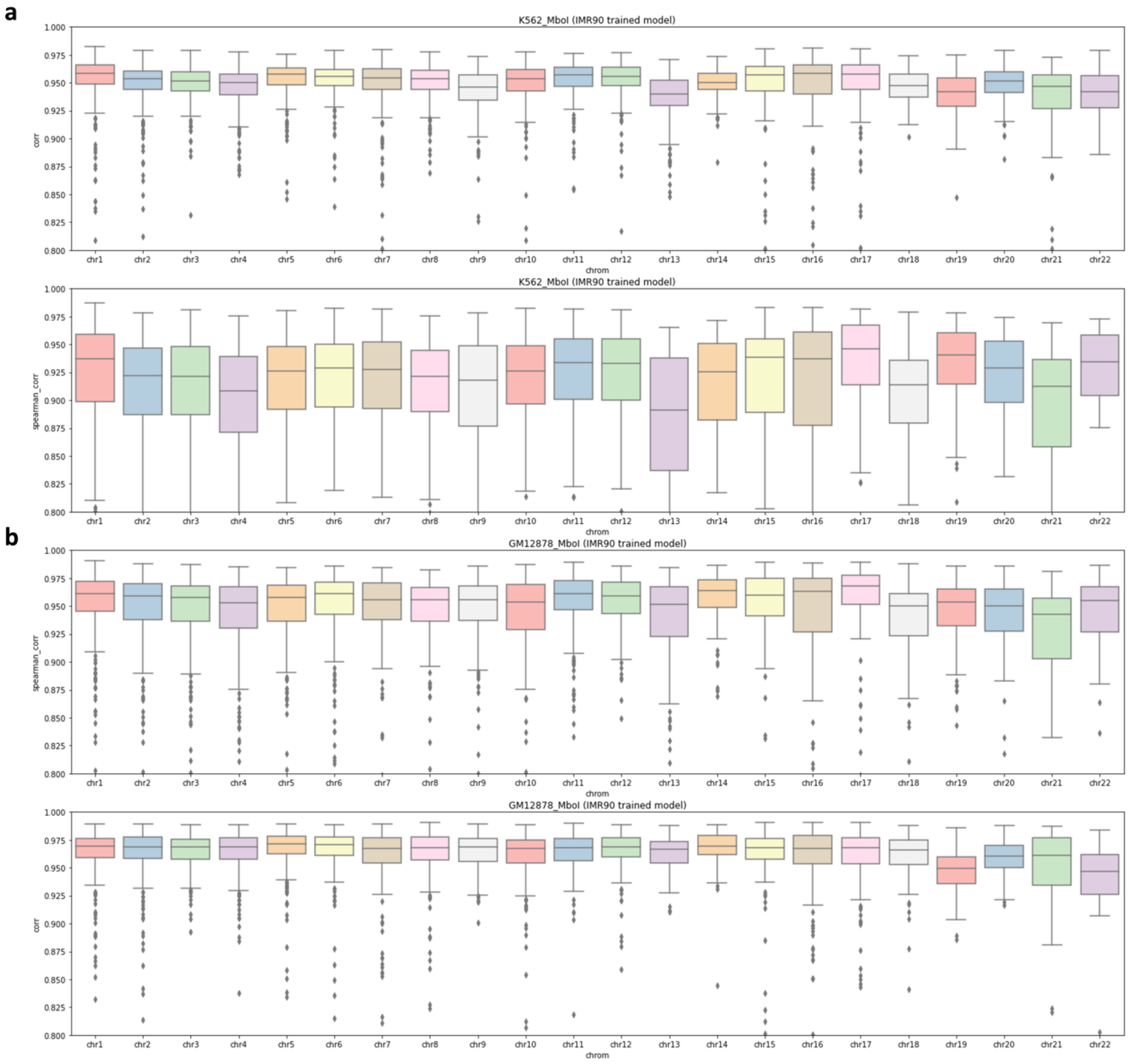

Supplementary Figure 3 Cross-cell Type Prediction Performance of IMR90-MboI Trained Model.

a. Prediction performance on K562 cells using the model trained on IMR90-MboI: The upper row displays Pearson correlation coefficients, while the lower row presents Spearman correlation coefficients across all chromosomes (n=5,199). **b. Prediction performance on GM12878 cells using the model trained on IMR90-MboI**: The upper row shows Pearson correlation coefficients, and the lower row shows Spearman correlation coefficients across all chromosomes (n=5,243). This figure demonstrates the robustness of our model in cross-cell type prediction, maintaining consistent performance across different chromosomes in both K562 and GM12878 cells. The boxplot elements are defined as follows: center line represents the median; box limits indicate the upper and lower quartiles; whiskers extend to 1.5 times the interquartile range from the quartiles; and points denote outliers.

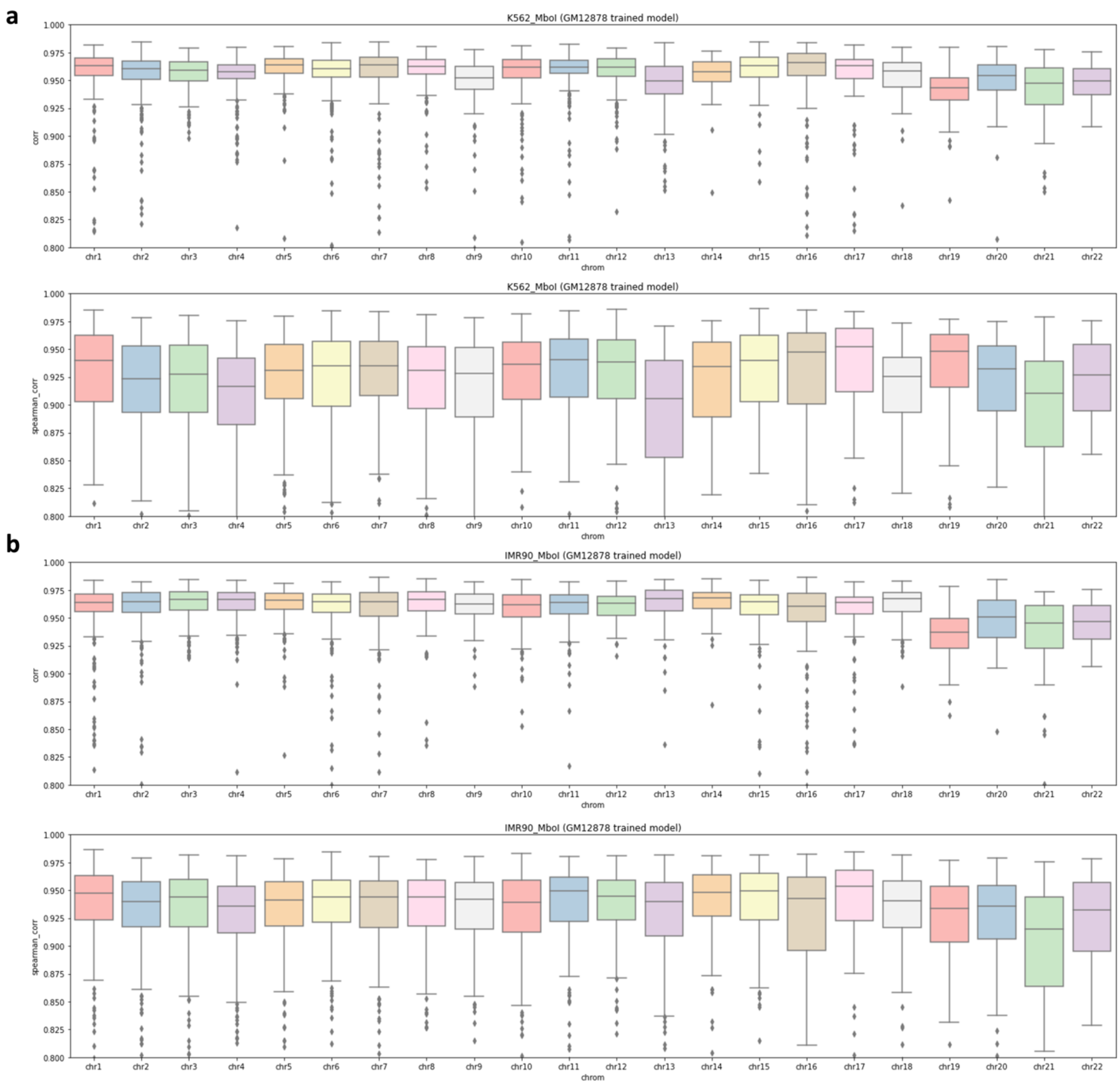

Supplementary Figure 4 GM12878-MboI trained model cross cell-type predictions performance.

a. Prediction performance on K562-MboI cells using the model trained on GM12878-MboI: The upper row shows Pearson correlation coefficients, and the lower row shows Spearman correlation coefficients across all chromosomes (n=5,220). The figure illustrates the cross-cell type prediction capability of our model trained on GM12878-MboI, showcasing consistent predictive performance when applied to both IMR90-MboI and K562-MboI cells. The boxplot elements are defined as follows: center line represents the median; box limits indicate the upper and lower quartiles; whiskers extend to 1.5 times the interquartile range from the quartiles; and points denote outliers. **b. Prediction performance on IMR90-MboI cells using the model trained on GM12878-MboI**: The upper row displays Pearson correlation coefficients, while the lower row presents Spearman correlation coefficients across all chromosomes (n=5,103).

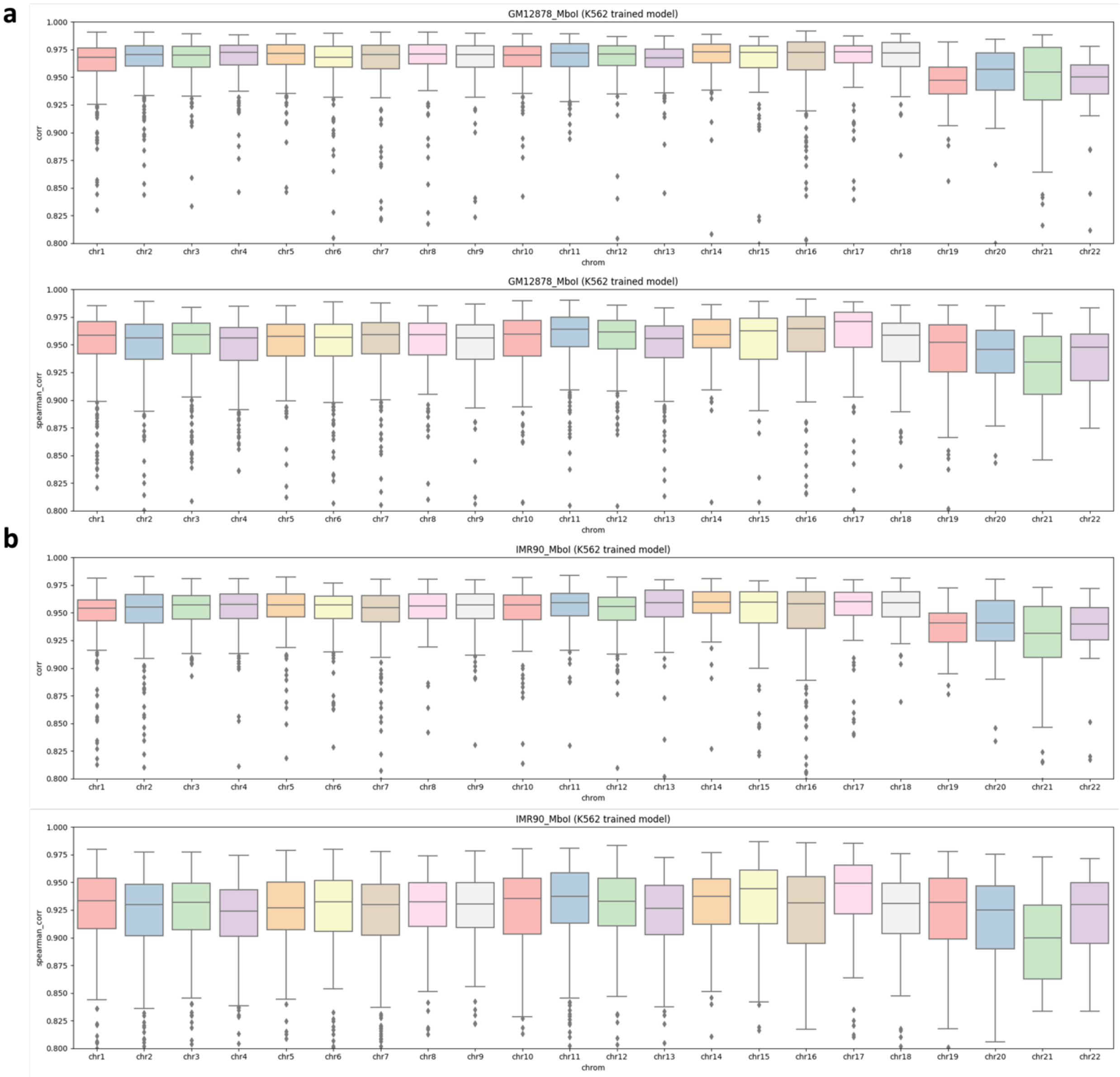

Supplementary Figure 5 Cross-cell Type Prediction Performance of K562-MboI Trained Model.

a. Prediction performance on GM12878-MboI cells using the model trained on K562-MboI: The upper row shows Pearson correlation coefficients, and the lower row shows Spearman correlation coefficients across all chromosomes (n=5,220). This figure demonstrates the cross-cell type prediction efficacy of our model trained on K562-MboI, with consistent performance across different chromosomes when predicting for IMR90-MboI and GM12878-MboI cells. The boxplot elements are defined as follows: center line represents the median; box limits indicate the upper and lower quartiles; whiskers extend to 1.5 times the interquartile range from the quartiles; and points denote outliers. **b. Prediction performance on IMR90-MboI cells using the model trained on K562-MboI:** The upper row displays Pearson correlation coefficients, while the lower row presents Spearman correlation coefficients across all chromosomes (n=5,243).

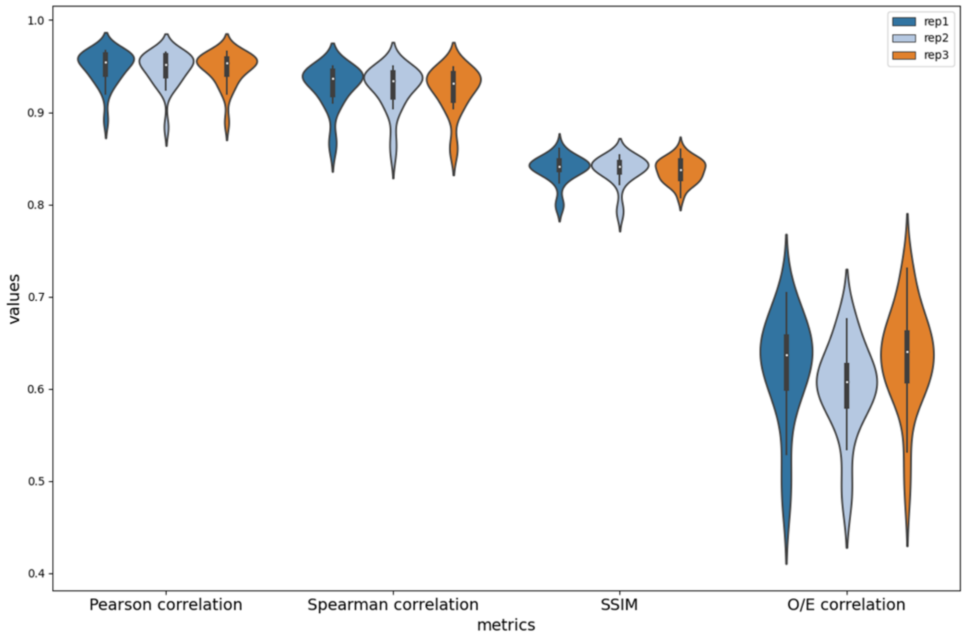

Supplementary Figure 6 EpiVerse shows high reproducibility.

After three retraining runs, EpiVerse's performance remained highly consistent on four evaluation metrics (n=5,243).

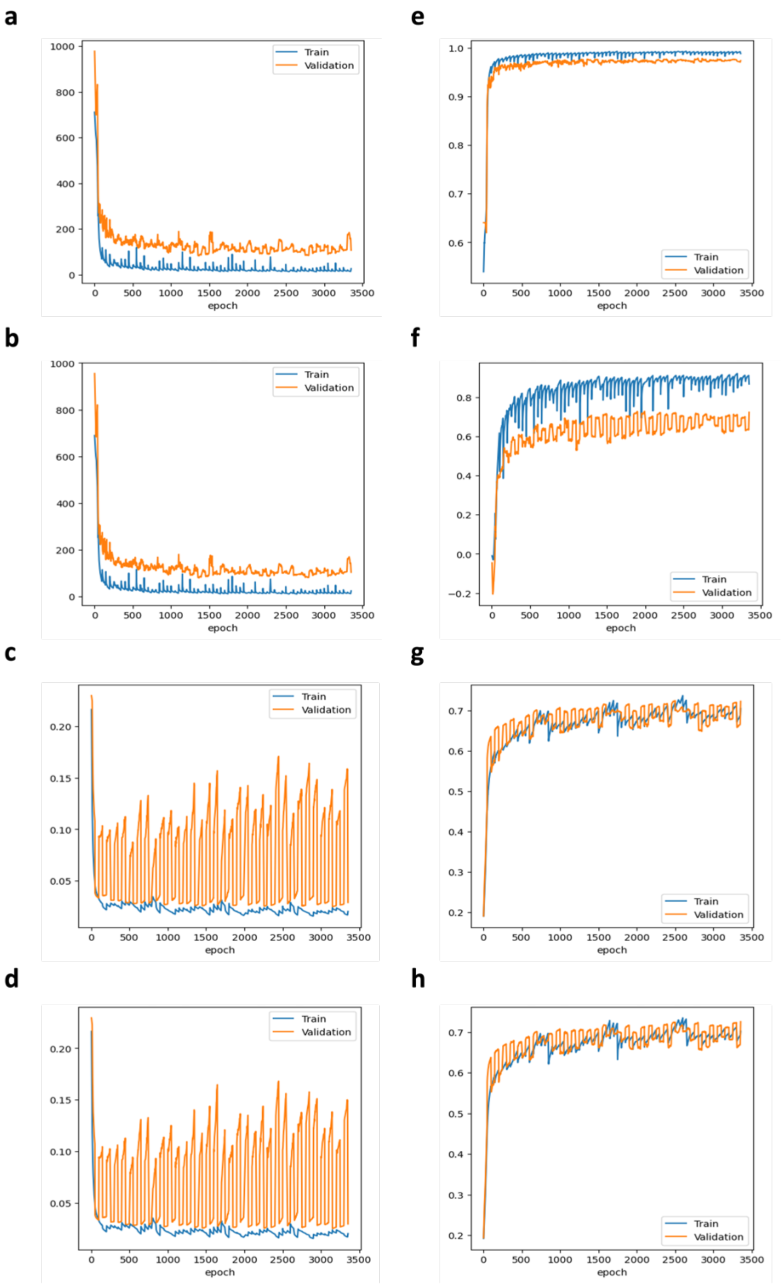

Supplementary Figure 7 EpiVerse Training Convergence and Performance Metrics in IMR90.

a. Epoch Loss: Shows overall loss reduction for training and validation. **b. Hi-C Loss:** Demonstrates the Hi-C loss convergence during training. **c. Bin1 ChromHMM State Loss:** Tracks the loss for Bin1 ChromHMM state prediction. **d. Bin2 ChromHMM State Loss:** Tracks the loss for Bin2 ChromHMM state prediction. **e. Hi-C Weighted Pearson Correlation:** Correlation between predicted and actual Hi-C data. **f. Hi-C Weighted Distance-Stratified Pearson Correlation:** Measures distance-stratified Pearson correlation between predicted and actual Hi-C data. **g. Bin1 ChromHMM State F1-Score:** Indicates precision and recall balance for Bin1. **h. Bin2 ChromHMM State F1-Score:** Indicates precision and recall balance for Bin2.

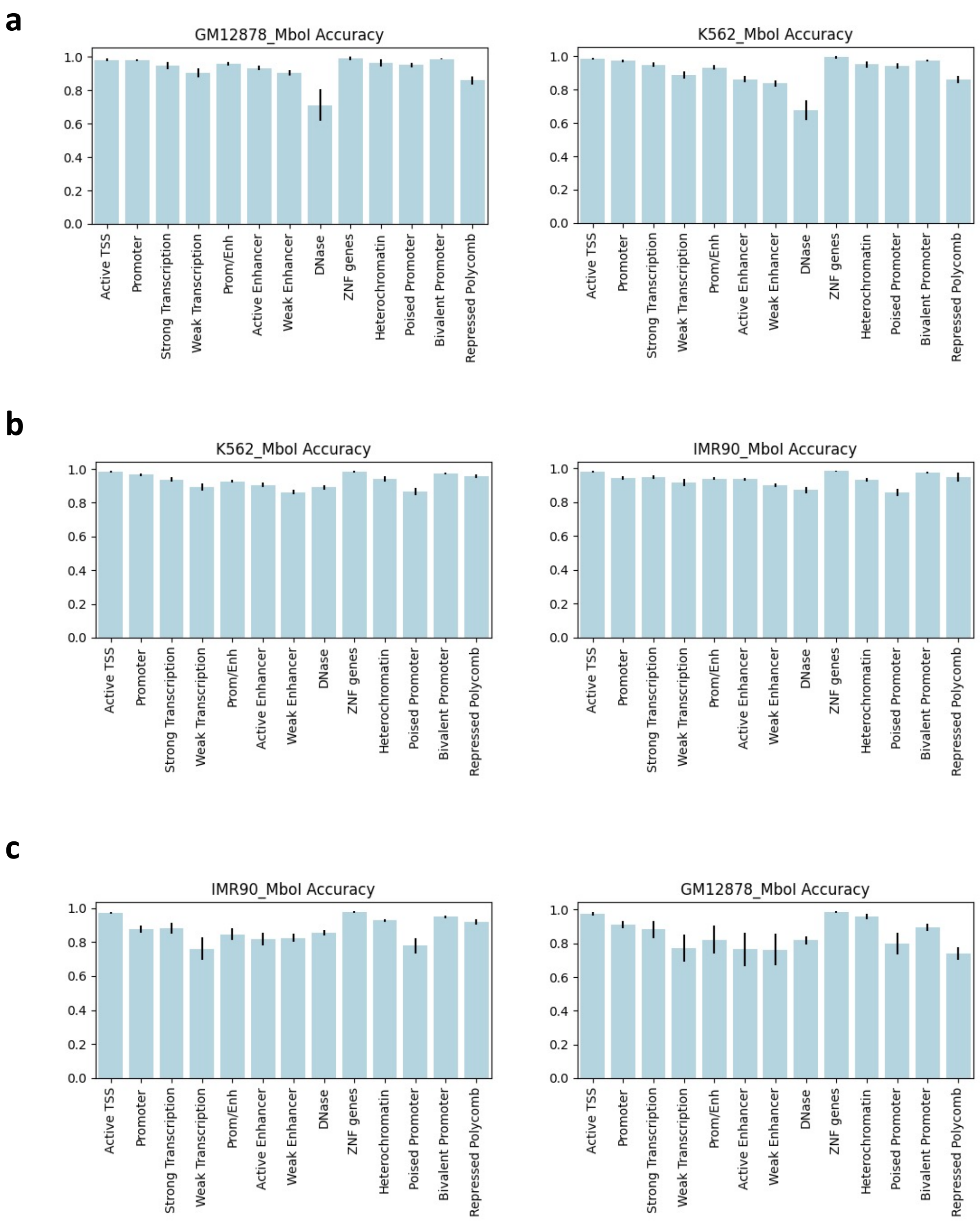

Supplementary Figure 8 ChromHMM Performance.

a. ChromHMM Prediction Accuracy of IMR90-MboI Trained Model: The bar chart compares the prediction accuracy of various chromatin states using the model trained on IMR90-MboI data across different cell lines (n=10,159). **b. ChromHMM Prediction Accuracy of GM12878-MboI Trained Model:** This bar chart shows the accuracy of ChromHMM state predictions using the GM12878-MboI trained model (n=10,159), demonstrating its performance across various cell lines. **c. ChromHMM Prediction Accuracy of K562-MboI Trained Model:** The bar chart illustrates the prediction accuracy for different chromatin states using the K562-MboI trained model (n=10,159), reflecting its predictive capability across diverse cellular contexts. Error bars indicate the standard deviations of accuracy.

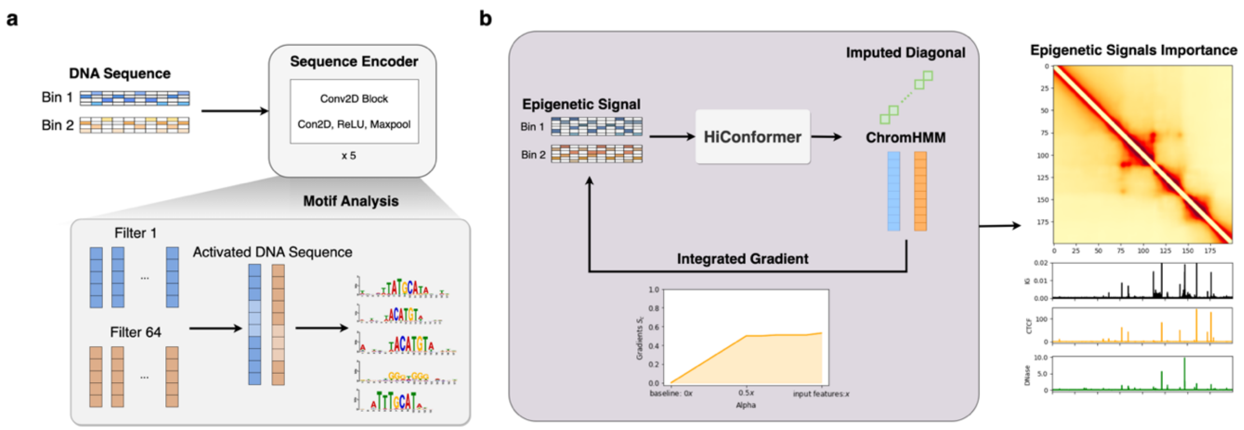

Supplementary Figure 9 HiConformer model interpretability methods.

a. DNA Motif Analysis via first layer of CNN activations: Illustration of DNA motif analysis using activations from the first layer of a Convolutional Neural Network, involving filtering and constructing Position Weight Matrices to identify consensus sequences. **b. Integrated Gradient (IG) Analysis of Epigenetic Signals:** Graph representing the IG assessment for a comprehensive set of epigenetic signals, highlighting their importance in the chromatin structure.

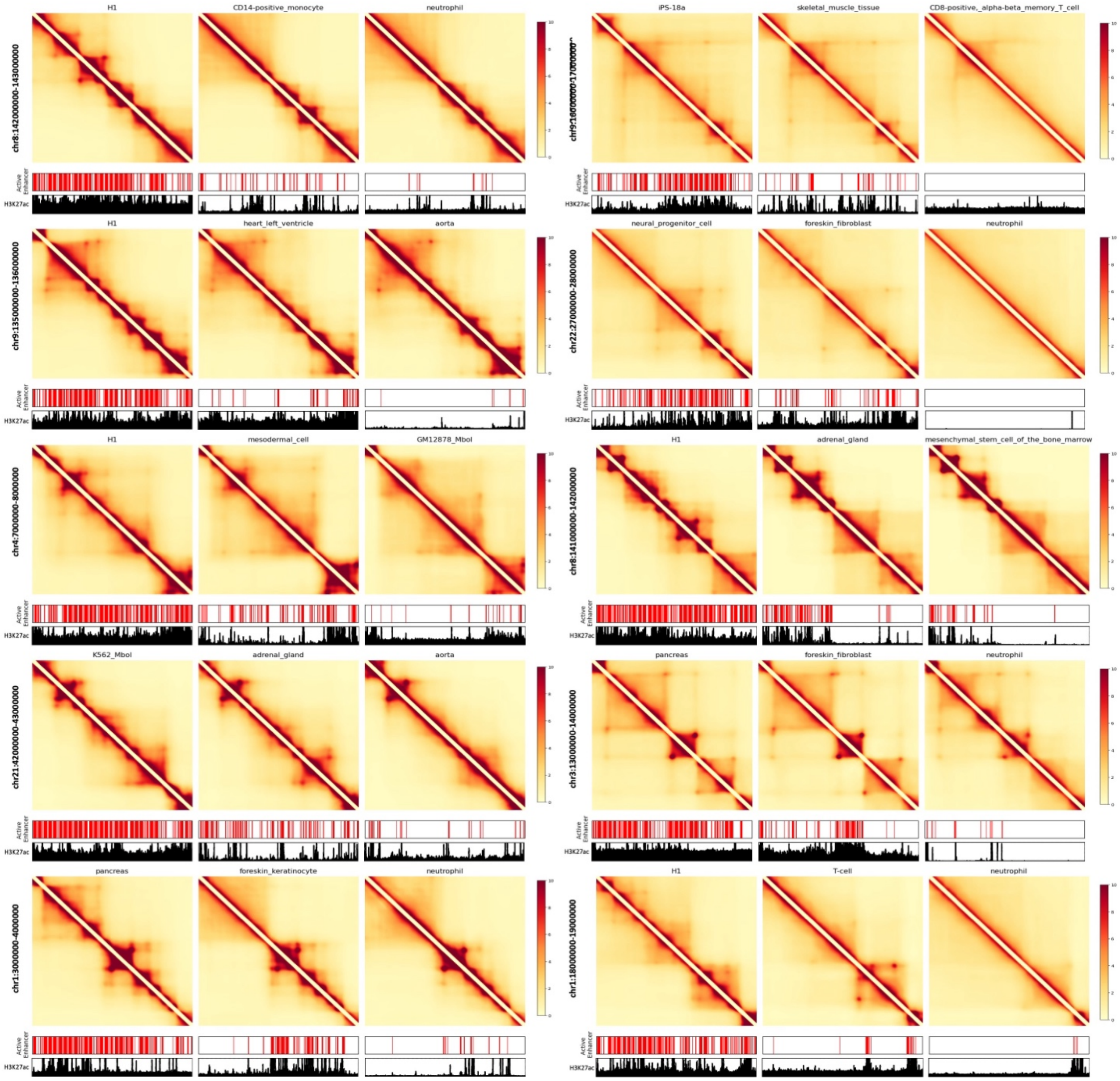

Supplementary Figure 10 Comparative Visualization of Enhancer Variation Regions.

Each panel presents Hi-C contact maps for selected genomic regions, showcasing the distribution of active enhancers alongside H3K27ac signal tracks. Progressing from left to right, the panels illustrate a decline in the count of active enhancers, paralleled by a corresponding decrease in Hi-C interaction frequencies.

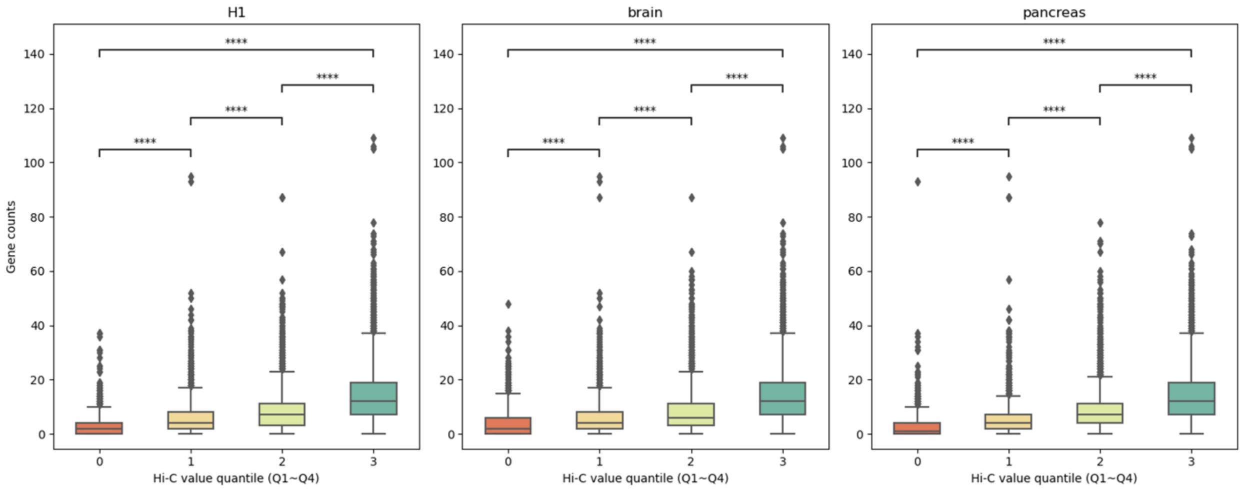

Supplementary Figure 11 Boxplot of Hi-C Value Quantile vs. Gene Counts.

Boxplot compares the distribution of Hi-C interaction values across four quantiles to the counts of gene counts (n=11,152), highlighting a trend that suggests a relationship between chromatin interaction frequency and gene counts. The two-sided Mann-Whitney test was conducted, with significance indicated by asterisks: *$p < 0.05$, **$p < 0.01$, ***$p < 0.001$, and ****$p < 0.0001$. Pairwise comparisons between groups for three tissues: H1 (0 vs. 1, $p=3.748 \times 10^{-136}$; 1 vs. 2, $p=4.565 \times 10^{-61}$; 2 vs. 3, $p=7.315 \times 10^{-152}$; 0 vs. 3, p=0), brain (0 vs. 1, $p=9.454 \times 10^{-73}$; 1 vs. 2, $p=2.498 \times 10^{-45}$; 2 vs. 3, $p=1.676 \times 10^{-140}$; 0 vs. 3, p=0), pancreas (0 vs. 1, $p=2.089 \times 10^{-147}$; 1 vs. 2, $p=5.797 \times 10^{-100}$; 2 vs. 3, $p=9.984 \times 10^{-146}$; 0 vs. 3, p=0). The boxplot elements are defined as follows: center line represents the median; box limits indicate the upper and lower quartiles; whiskers extend to 1.5 times the interquartile range from the quartiles; and points denote outliers.

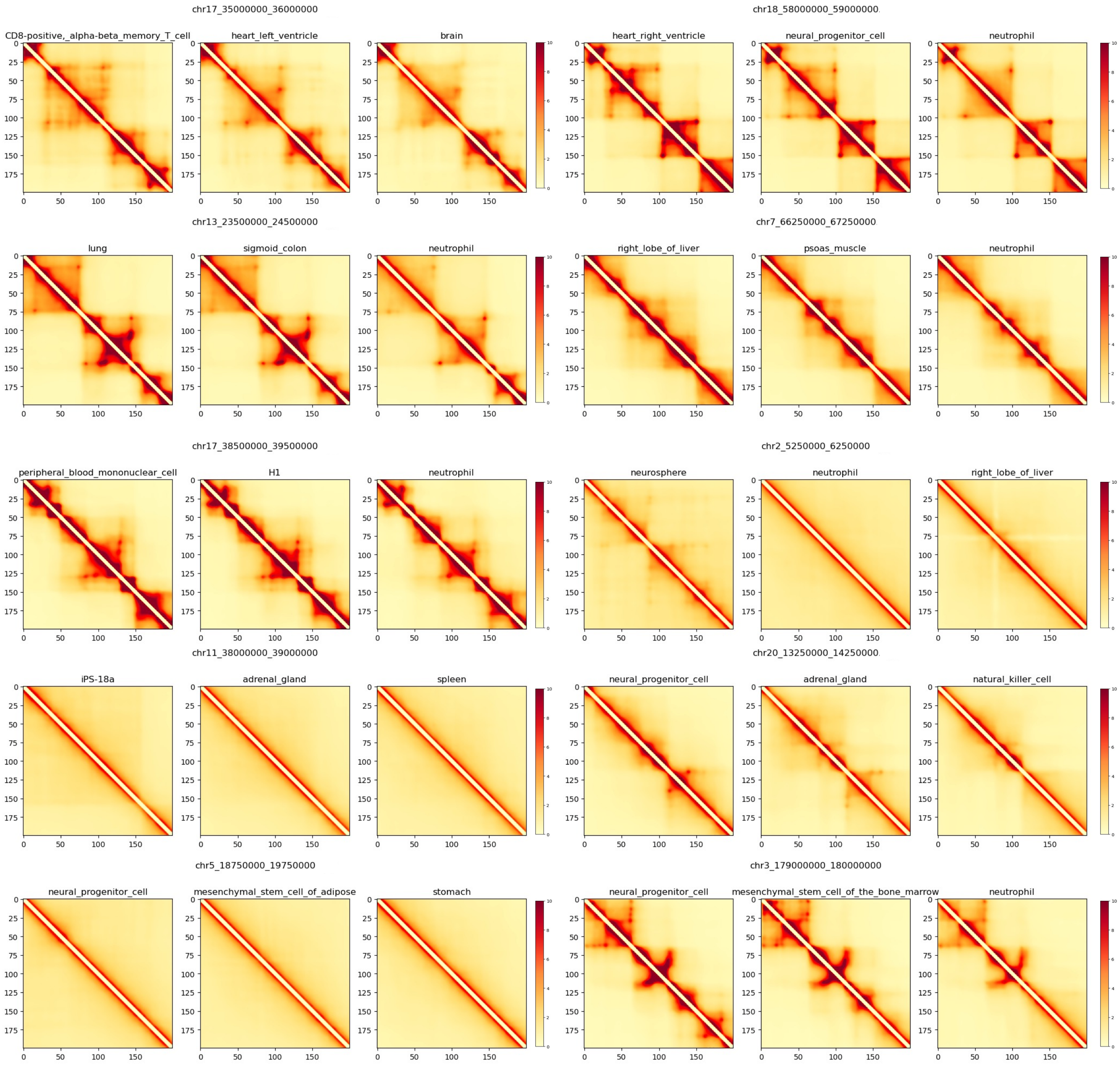

Supplementary Figure 12 Comparative Visualization of Hi-C Variation Regions.

Each panel presents Hi-C contact maps for selected genomic regions, showcasing the variation regions between each tissue. Each tissue has its unique chromatin structure landscape.

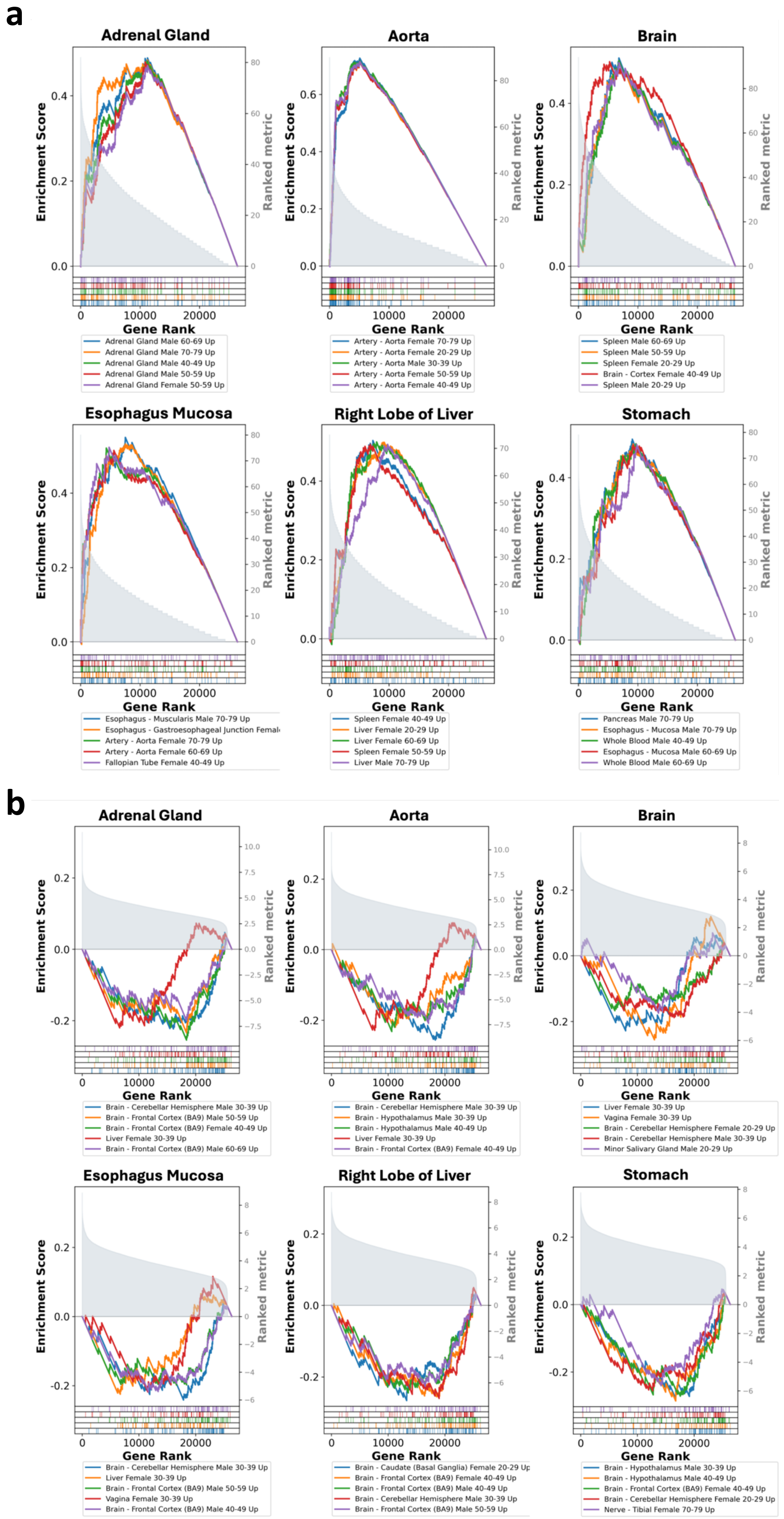
a
Adrenal Gland
Aorta
Brain
Enrichment Score
Ranked metric
Gene Rank
Adrenal Gland Male 60-69 Up
Adrenal Gland Male 70-79 Up
Adrenal Gland Male 40-49 Up
Adrenal Gland Male 50-59 Up
Adrenal Gland Female 50-59 Up
Artery - Aorta Female 70-79 Up
Artery - Aorta Female 20-29 Up
Artery - Aorta Male 30-39 Up
Artery - Aorta Female 50-59 Up
Artery - Aorta Female 40-49 Up
Spleen Male 60-69 Up
Spleen Male 50-59 Up
Spleen Female 20-29 Up
Brain - Cortex Female 40-49 Up
Spleen Male 20-29 Up
Esophagus Mucosa
Right Lobe of Liver
Stomach
Esophagus - Muscularis Male 70-79 Up
Esophagus - Gastroesophageal Junction Femal
Artery - Aorta Female 70-79 Up
Artery - Aorta Female 60-69 Up
Fallopian Tube Female 40-49 Up
Spleen Female 40-49 Up
Liver Female 20-29 Up
Liver Female 60-69 Up
Spleen Female 50-59 Up
Liver Male 70-79 Up
Pancreas Male 70-79 Up
Esophagus - Mucosa Male 70-79 Up
Whole Blood Male 40-49 Up
Esophagus - Mucosa Male 60-69 Up
Whole Blood Male 60-69 Up
b
Adrenal Gland
Aorta
Brain
Brain - Cerebellar Hemisphere Male 30-39 Up
Brain - Frontal Cortex (BA9) Male 50-59 Up
Brain - Frontal Cortex (BA9) Female 40-49 Up
Liver Female 30-39 Up
Brain - Frontal Cortex (BA9) Male 60-69 Up
Brain - Cerebellar Hemisphere Male 30-39 Up
Brain - Hypothalamus Male 30-39 Up
Brain - Hypothalamus Male 40-49 Up
Liver Female 30-39 Up
Brain - Frontal Cortex (BA9) Female 40-49 Up
Liver Female 30-39 Up
Vagina Female 30-39 Up
Brain - Cerebellar Hemisphere Female 20-29 Up
Brain - Cerebellar Hemisphere Male 30-39 Up
Minor Salivary Gland Male 20-29 Up
Esophagus Mucosa
Right Lobe of Liver
Stomach
Brain - Cerebellar Hemisphere Male 30-39 Up
Liver Female 30-39 Up
Brain - Frontal Cortex (BA9) Male 50-59 Up
Vagina Female 30-39 Up
Brain - Frontal Cortex (BA9) Male 40-49 Up
Brain - Caudate (Basal Ganglia) Female 20-29 Up
Brain - Frontal Cortex (BA9) Female 40-49 Up
Brain - Frontal Cortex (BA9) Male 40-49 Up
Brain - Cerebellar Hemisphere Male 30-39 Up
Brain - Frontal Cortex (BA9) Male 50-59 Up
Brain - Hypothalamus Male 30-39 Up
Brain - Hypothalamus Male 40-49 Up
Brain - Frontal Cortex (BA9) Female 40-49 Up
Brain - Cerebellar Hemisphere Female 20-29 Up
Nerve - Tibial Female 70-79 Up

Supplementary Figure 13 Comparative Analysis of Tissue-Specific Gene Identification by Promoters and Active Enhancers Using Gene Set Enrichment Analysis (GSEA)[3].

a. Active enhancer-based GSEA plots demonstrate tissue-specific gene expression patterns identified across various tissues. The criterion for an active enhancer's effectiveness in pinpointing tissue-specific gene expression is its inclusion in the top 5 GSEA ranks for the target tissue. The analysis highlights that active enhancers are successful in identifying the correct tissue-specific gene expression profiles in 12 out of the 19 tissues when this criterion is met, specifically in the adrenal gland, aorta, B cells, brain, colonic mucosa, esophagus muscularis mucosa, esophagus squamous epithelium, heart (left ventricle and right ventricle), natural killer cells, psoas muscle, and the right lobe of liver.

b. Promoter-based GSEA plots display tissue-specific gene expression identification across the same set of tissues with active enhancer-based GSEA plots. Promoters only successfully identifiy tissue-specific gene expression profiles in the brain and right lobe of the liver.

Labels such as “70-79 Up” refer to the age range of individuals in the GTEx dataset (e.g., 70-79 years), with "Up" indicating upregulated genes in that age group compared to baseline.

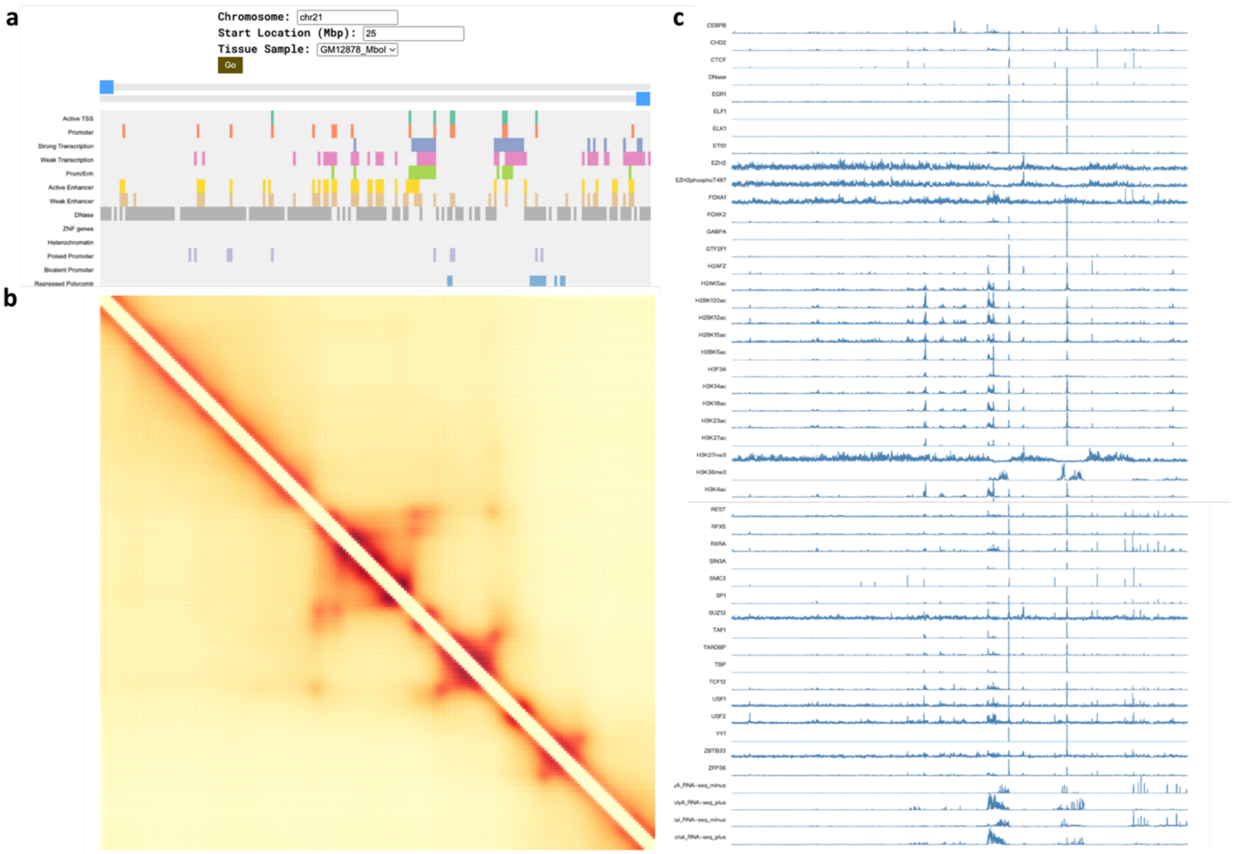

Supplementary Figure 14 Virtual Epigenetic Landscape Viewer of EpiVerse Viewer.

a. User Interface and Chromatin State Visualization: Our webserver offers an intuitive interface allowing users to specify the chromosome and the starting location in megabase pairs (Mbp) to examine each 1M region of interest in detail. An interactive scrollbar is integrated to facilitate focused exploration within specified genomic locales. Accompanying the scrollbar, the server presents a imputation ChromHMM visualization that annotates the chromatin states within the selected 1M region, enabling insights into chromatin organization. **b. Hi-C Contact Map Visualization:** The EpiVerse Viewer renders high-resolution, imputed Hi-C interaction matrices, providing a detailed view of chromosomal interactions. This visualization is instrumental in identifying and studying the structural configuration of the genome. **c. Epigenetic Signals:** In addition to chromatin interactions, our server provides imputed epigenetic signals as inferred by the Avocado model. This comprehensive representation elucidates the intricate interplay between epigenetic modifications and chromatin architecture, offering a valuable resource for investigating the regulatory mechanisms underpinning genomic structure and function.

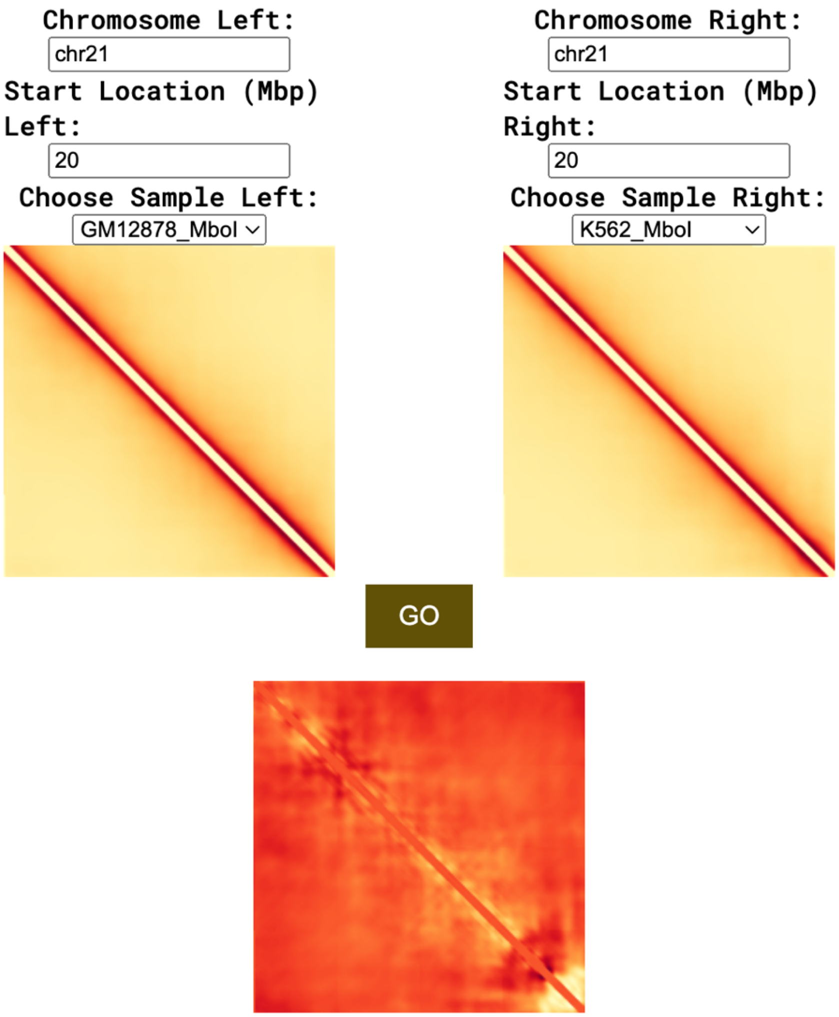

Supplementary Figure 15 Comparison Function of EpiVerse Viewer.

EpiVerse Viewer's comparison feature simplifies the examination process by enabling direct juxtaposition between two tissues, streamlining the task of analyzing divergent Hi-C datasets.

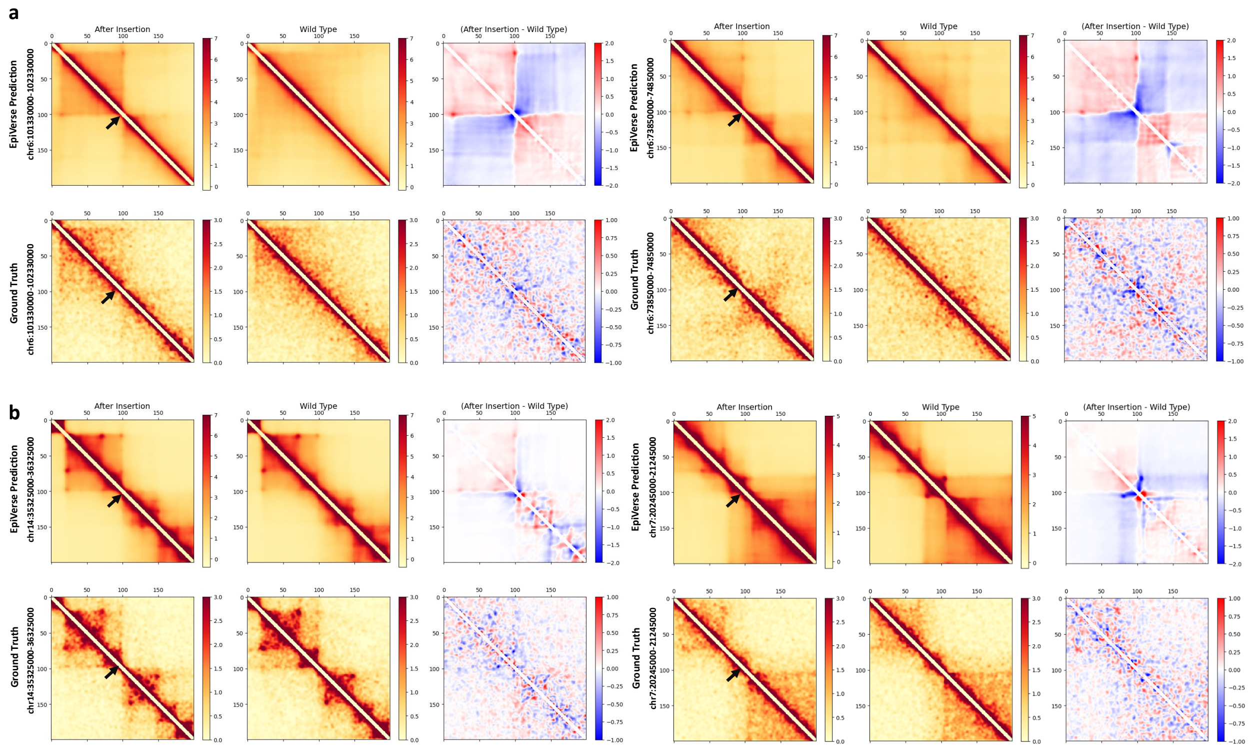

Supplementary Figure 16 *in silico* insertion Hi-C experiment[2].

The figure shows both the predicted and observed contact matrices for the DNA insertion experiment. The original study was conducted in the HAP1 cell line, which is unavailable in the Avocado pre-trained model. Therefore, imputed H1 cell line data was used to demonstrate similar boundary-forming effects in our predictions. Despite the differences in cell lines, the comparison illustrates the congruence between EpiVerse's in silico perturbations and real Hi-C data, showing new chromatin contacts and reinforced domain boundaries. Arrows indicate insertion sites. **a. De novo Contact Formation:** This figure illustrates the creation of new chromatin contacts following insertion of a 2-kb DNA sequence at a tissue-invariant domain boundary. The heatmap comparison clearly shows the emergence of interactions that were not present prior to the insertion event. **b. Additive Boundary Strengthening:** This figure depicts the reinforcement of existing chromatin boundaries post-insertion. The heatmaps contrast the chromatin interaction patterns before and after the insertion, highlighting the enhanced boundary definition as a result of the experiment.

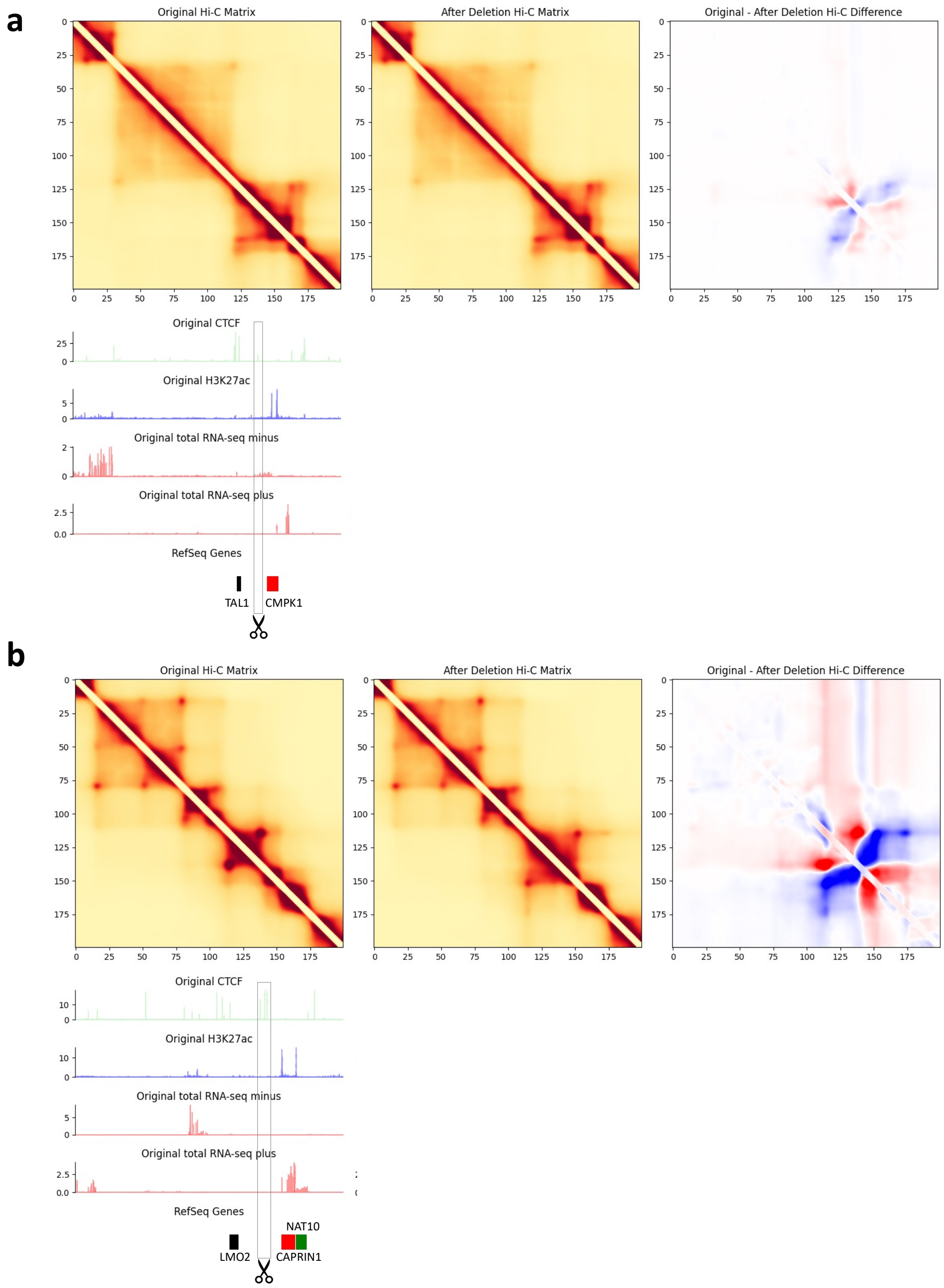

Supplementary Figure 17 ***In silico*** **deletion Hi-C experiment[1].**

a. TAL1 Oncogene Activation: This figure presents the consequences of deletion near the TAL1 insulated boundary. The heatmaps compare chromatin structures pre- and post-deletion, with arrows highlighting the activation of the TAL1 oncogene due to the deletion event. **b. LMO2 Oncogene Activation:** The figure demonstrates the impact of a targeted deletion near the LMO2 insulated boundary, showing the resultant activation of the LMO2 oncogene. The heatmaps before and after deletion illustrate the changes in chromatin interaction patterns, with arrows indicating the specific areas of alteration.

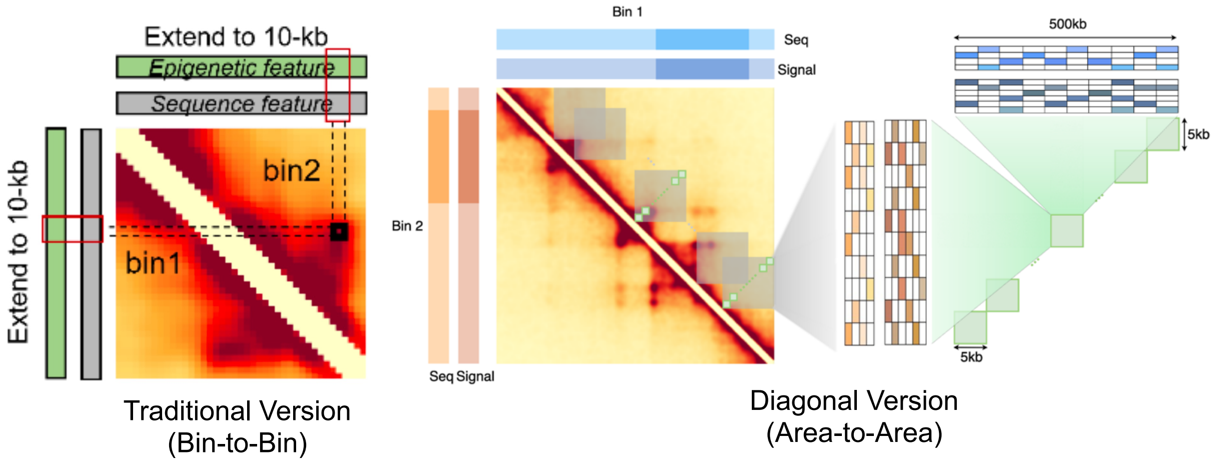

Supplementary Figure 18 Diagonal Extraction Algorithm for expanding the model's receptive field. This figure illustrates the Diagonal Extraction Algorithm, which expands the model's receptive field by incorporating neighboring information for chromatin structure prediction. Unlike traditional approaches (Bin-to-Bin), where each Hi-C contact is predicted using only the features at that specific contact point, this method extracts entire diagonals from the ground truth Hi-C matrices, allowing each feature to leverage neighboring genomic interactions (Area-to-Area).

References

1 Hnisz, D. *et al.* Activation of proto-oncogenes by disruption of chromosome neighborhoods. *Science* **351**, 1454-1458 (2016).

2 Zhang, D. *et al.* Alteration of genome folding via contact domain boundary insertion. *Nature genetics* **52**, 1076-1087 (2020).

3 Consortium, G. The GTEx Consortium atlas of genetic regulatory effects across human tissues. *Science* **369**, 1318-1330 (2020).

4 Bailey, T. L. *et al.* MEME SUITE: tools for motif discovery and searching. *Nucleic acids research* **37**, W202-W208 (2009).
