# Supplementary material for: Unveiling chromatin dynamics with virtual epigenome
Source: Nat Commun. 2025 Apr 12;16:3491. doi: 10.1038/s41467-025-58481-3 (PMC11993739; doi:10.1038/s41467-025-58481-3)
Supplement: Supplementary file 2 — Description of Additional Supplementary Files [file 41467_2025_58481_MOESM2_ESM.pdf]

Description of Additional Supplementary Files

Supplementary Data 1: 71 selected (high-quality) tracks of epigenetic signals in Avocado.

Supplementary Data 2: Detailed description of training/evaluation tissues data.

Supplementary Data 3: HiConformer sequence encoder motif matching by TOMTOM4. This table shows the top five matching motifs for each filter in the HiConformer sequence encoder.

Supplementary Data 4: chromosome 21 differential genes between GM12878 and K562 using Integrated Gradient (IG) with MSigDB C4 computational gene sets.

Supplementary Data 5: whole genome differential genes between GM12878 and K562 using Integrated Gradient (IG) with MSigDB C6 oncogenic signature gene sets.

Supplementary Data 6: whole genome differential genes between GM12878 and K562 using Integrated Gradient (IG) with MSigDB C4 computational gene sets.

Supplementary Data 7: A Selection of 39 Representative Tissues Sampled from the Roadmap Tissue/Cell Type Groups.

Supplementary Data 8 Whole Genome Inference time comparison between EpiVerse, C.Origami, and Orca. We compare the running times of the three models on a NVIDIA Tesla V100 GPU and 32 cores of an Intel(R) Xeon(R) Gold 6154 CPU. HiC-Reg was excluded due to its CPU-only implementation.
