# Supplementary material for: Unveiling chromatin dynamics with virtual epigenome
Source: Nat Commun. 2025 Apr 12;16:3491. doi: 10.1038/s41467-025-58481-3 (PMC11993739; doi:10.1038/s41467-025-58481-3)
Supplement: Supplementary file 11 — Reporting Summary [file 41467_2025_58481_MOESM11_ESM.pdf]

nature portfolio

Corresponding author(s): Jui-Hung Hung
Last updated by author(s): Mar 8, 2025

Reporting Summary

Nature Portfolio wishes to improve the reproducibility of the work that we publish. This form provides structure for consistency and transparency in reporting. For further information on Nature Portfolio policies, see our Editorial Policies and the Editorial Policy Checklist.

Statistics

For all statistical analyses, confirm that the following items are present in the figure legend, table legend, main text, or Methods section.

n/a	Confirmed	
☐	☒	The exact sample size (*n*) for each experimental group/condition, given as a discrete number and unit of measurement
☒	☐	A statement on whether measurements were taken from distinct samples or whether the same sample was measured repeatedly
☐	☒	The statistical test(s) used AND whether they are one- or two-sided<br>*Only common tests should be described solely by name; describe more complex techniques in the Methods section.*
☒	☐	A description of all covariates tested
☒	☐	A description of any assumptions or corrections, such as tests of normality and adjustment for multiple comparisons
☐	☒	A full description of the statistical parameters including central tendency (e.g. means) or other basic estimates (e.g. regression coefficient) AND variation (e.g. standard deviation) or associated estimates of uncertainty (e.g. confidence intervals)
☐	☒	For null hypothesis testing, the test statistic (e.g. *F*, *t*, *r*) with confidence intervals, effect sizes, degrees of freedom and *P* value noted<br>*Give P values as exact values whenever suitable.*
☒	☐	For Bayesian analysis, information on the choice of priors and Markov chain Monte Carlo settings
☒	☐	For hierarchical and complex designs, identification of the appropriate level for tests and full reporting of outcomes
☒	☐	Estimates of effect sizes (e.g. Cohen's *d*, Pearson's *r*), indicating how they were calculated

Our web collection on statistics for biologists contains articles on many of the points above.

Software and code

Policy information about availability of computer code

Data collection

We used our own customized code to download the hg38 reference genome from UCSC, Avocado's pre-impute epigenetic signals, Hi-C data from 3DIV, and ChromHMM's histone state data from the Roadmap Epigenomics Project portals. All scripts and code used for data collection are available in our GitHub repository: https://github.com/jhhung/EpiVerse.

Data analysis

For data analysis, we employ a customized pipeline to process DNA sequences, epigenetic signals, Hi-C data, and ChromHMM histone state annotations. Our workflow consists of the following steps:

DNA Sequence Processing: We obtain the hg38 reference genome from UCSC using our custom script (HiConformer_ref_crawler.py).
Epigenetic Signal Extraction: We generate custom imputed epigenetic signals using Avocado. The target tissue is specified based on metadata within the Avocado directory, and the corresponding data is retrieved using Avocado_preimpute_crawler.py.

Hi-C Data Retrieval and Processing: We acquire Hi-C interaction data from 3DIV, selecting the appropriate tissue and downloading the dataset via HiConformer_3DIV_crawler.py. To refine the analysis, we perform peak calling using HICCUPS (Version 0.3.5).
ChromHMM Histone State Annotation: We identify the relevant Epigenome ID (EID) for the target tissue and download the ChromHMM histone state data using HiConformer_ChromHMM_crawler.py.

All scripts used for data processing are executed within the pipelines directory after activating the appropriate environment (conda activate HiConformer). The complete data analysis pipeline, including scripts and instructions, is available in our GitHub repository: https://github.com/jhhung/EpiVerse.

For manuscripts utilizing custom algorithms or software that are central to the research but not yet described in published literature, software must be made available to editors and reviewers. We strongly encourage code deposition in a community repository (e.g. GitHub). See the Nature Portfolio guidelines for submitting code & software for further information.

Data

Policy information about availability of data

All manuscripts must include a data availability statement. This statement should provide the following information, where applicable:

- Accession codes, unique identifiers, or web links for publicly available datasets
- A description of any restrictions on data availability
- For clinical datasets or third party data, please ensure that the statement adheres to our policy

The GRCh38/hg38 reference genome used in this study was downloaded from the UCSC database https://hgdownload.soe.ucsc.edu/goldenPath/hg38/bigZips. The Hi-C data for IMR90, K562, and GM12878 cell lines with the MboI restriction enzyme used for training models have been obtained from the 3DIV database http://3div.kr/download. The ChromHMM data for IMR90 (E017), GM12878 (E116), and K562 (E123), based on the 25-state, 12-mark chromatin state model from the ROADMAP Epigenomics project, were also used for training models and are available at the ROADMAP Epigenomics portal https://egg2.wustl.edu/roadmap/web_portal/index.html. High-quality imputed epigenetic signals from Avocado are listed in Table 1, and the ENCODE accession IDs for each track can be found in the following repository: https://github.com/jhhung/EpiVerse/blob/main/Avocado/Avocado_metadata.csv. The In silico perturbational Hi-C datasets perturbational Hi-C datasets used in this study are available from the NCBI GEO database under accession codes GSE137374 [https://www.ncbi.nlm.nih.gov/geo/query/acc.cgi?acc=GSE137374], GSE68976 [https://www.ncbi.nlm.nih.gov/geo/query/acc.cgi?acc=GSE68976], and GSE149103 [https://www.ncbi.nlm.nih.gov/geo/query/acc.cgi?acc=GSE149103]. The pretrained model weights for IMR90, K562, GM12878, and EpiVerse-imputed ChromHMM data across 41 tissues/cell lines generated in this study are available at https://zenodo.org/records/13759557. For any requests requiring more complete data, please contact juihunghung@gmail.com.

Research involving human participants, their data, or biological material

Policy information about studies with human participants or human data. See also policy information about sex, gender (identity/presentation), and sexual orientation and race, ethnicity and racism.

Reporting on sex and gender	This research involved no human subject. Not collected.
Reporting on race, ethnicity, or other socially relevant groupings	This research involved no human subject. Not collected.
Population characteristics	see above.
Recruitment	None recruited.
Ethics oversight	No approval needed.

Note that full information on the approval of the study protocol must also be provided in the manuscript.

Field-specific reporting

Please select the one below that is the best fit for your research. If you are not sure, read the appropriate sections before making your selection.

☒ Life sciences ☐ Behavioural & social sciences ☐ Ecological, evolutionary & environmental sciences

For a reference copy of the document with all sections, see nature.com/documents/nr-reporting-summary-flat.pdf

Life sciences study design

All studies must disclose on these points even when the disclosure is negative.

Sample size	In our study, the samples correspond to unit bins across the entire genome within a specific genomic region. The sample size is determined by collecting all possible such bins within the designated area.
Data exclusions	No data excluded.
Replication	We replicated the training process three times and found that the performance was consistently reliable across all runs, which we have shown in Supplementary Figure 6.
Randomization	Since our analysis includes all available samples within the designated genomic regions, no randomization was performed.
Blinding	Blinding is not relevant to our study because our analysis is based on computational processing of genomic datasets rather than subjective assessments or experimental conditions that involve human or observer bias. The data (e.g., DNA sequences, epigenetic signals, Hi-C interactions) are objectively collected and analyzed using predefined algorithms, eliminating the need for blinding.

Reporting for specific materials, systems and methods

We require information from authors about some types of materials, experimental systems and methods used in many studies. Here, indicate whether each material, system or method listed is relevant to your study. If you are not sure if a list item applies to your research, read the appropriate section before selecting a response.

Materials & experimental systems

n/a	Involved in the study
☒	☐ Antibodies
☒	☐ Eukaryotic cell lines
☒	☐ Palaeontology and archaeology
☒	☐ Animals and other organisms
☒	☐ Clinical data
☒	☐ Dual use research of concern
☒	☐ Plants

Methods

n/a	Involved in the study
☒	☐ ChIP-seq
☒	☐ Flow cytometry
☒	☐ MRI-based neuroimaging

Plants

Seed stocks	This research involved no plant.
Novel plant genotypes	This research involved no plant.
Authentication	This research involved no plant.
